# Real-world insights from acute management of potassium disorders in diabetic ketoacidosis

**DOI:** 10.3389/fendo.2025.1669400

**Published:** 2025-11-03

**Authors:** Yue Zhang, Jing Li, Shanshan Deng, Yongjing Zhang, Manli Guo, Fangfang Jiang, Lingguang Luo, Yanqing He, Shaogang Ma, Yigen Peng

**Affiliations:** ^1^ Department of Clinical Laboratory, the People’s Hospital of Laibin, Laibin, China; ^2^ Department of Endocrinology and Metabolism, Suqian First Hospital, Suqian, Jiangsu, China; ^3^ Department of Endocrinology and Metabolism, Liuzhou People’s Hospital, Liuzhou, China; ^4^ Department of Endocrinology and Metabolism, the Third People’s Hospital of Bengbu, Bengbu, Anhui, China; ^5^ Department of Endocrinology and Metabolism, the Affiliated Suqian Hospital of XuZhou Medical University and Nanjing Drum Tower Hospital Group Suqian Hospital, Suqian, Jiangsu, China; ^6^ Department of Endocrinology and Metabolism, the Third Hospital Affiliated to Nanchang University, Nanchang, Jiangxi, China; ^7^ Department of Endocrinology and Metabolism, the People’s Hospital of Laibin, Laibin, China; ^8^ Emergency Department, the Affiliated Jiangning Hospital of Nanjing Medical University, Nanjiang, Jiangsu, China

**Keywords:** potassium disorders, diabetic ketoacidosis, treatment, evaluation, potassium replenishment

## Abstract

**Background:**

Diabetic ketoacidosis (DKA) is a severe hyperglycemic emergency characterized by metabolic acidosis and electrolyte disturbances. The optimal strategy for potassium replenishment in DKA remains incomplete. This study comprehensively characterized potassium disturbances in DKA and evaluated the effectiveness of potassium replenishment strategies, with a focus on the risk of hypokalemia during treatment.

**Methods:**

In this multicentre retrospective cohort study, we enrolled the consecutive DKA patients admitted to seven tertiary centres across eastern, central and western China (1 January 2021–31 December 2023). Demographics, biochemical parameters and daily potassium chloride (KCl) replenishment were extracted and evaluated. We used multivariable logistic regression to identify predictors of hypokalaemia during treatment, internally validated the model, and constructed a practical nomogram.

**Results:**

A total of 571 eligible subjects were included in the analysis. On admission, blood glucose, arterial pH, HCO_3_^-^, and electrolyte profiles were seriously deteriorated. Among the patients, 95 patients (16.6%) were hypokalemic, 352 (61.6%) normokalemic and 124 (21.7%) hyperkalemic. Hyperkalemia was more frequent in severe DKA and associated with renal impairment and the severity of DKA (*p* < 0.05). During treatment, 388 (67.9%) patients developed hypokalemia, the proportion rose to 73.6% among severe DKA cases. The occurrence of hypokalemia during treatment was independently associated with potassium concentration, HbA1c, and arterial pH at admission (*p* < 0.05). The statistical model predicted the risk of hypokalemia during treatment. A daily 6.0 g KCl supplement offered superior predictive efficacy for hypokalemia compared to lower doses throughout the treatment course.

**Conclusions:**

Potassium imbalances were highly prevalent in DKA. Although hyperkalemia was more common on admission, hypokalemia frequently developed during treatment. Daily 6.0 g KCl replenishment was superior to lower doses in predicting hypokalaemia. This study provided the full spectrum of potassium disorders in DKA and delivered an evidence-based, patient-specific replenishment framework.

## Introduction

Diabetic ketoacidosis (DKA) is a severe hyperglycemic emergency characterized by a triad of hyperglycemia, metabolic acidosis, and hyperketonemia. It commonly occurs in individuals with type 1 diabetes mellitus (T1D) and type 2 diabetes mellitus (T2D). DKA can trigger a cascade of internal disruptions, including acid-base imbalances, dehydration, and electrolyte disturbances. As the condition progresses, it may lead to severe complications (1–4).

An insulin deficiency, whether absolute or relative, combined with hyperglycemia and ketoacidosis, leads to the shift of potassium into the extracellular space. When blood glucose levels exceed the renal threshold, glycosuria is triggered, which in turn induces osmotic diuresis. The process results in dehydration and substantial loss of electrolytes (5). Electrolyte disturbances, particularly those involving potassium, are common in patients with DKA (3).

Potassium imbalances can manifest as cardiac dysrhythmias. Without potassium supplementation within 24 hours, the risk of death increases threefold. Severe hypokalemia can lead to a four-fold increase in the risk of unfavorable treatment outcomes (6, 7). Previous studies have shown the impact on electrolyte disorders and liver and kidney function (3, 8), whereas the effect of potassium replenishment has not been well characterized.

Given the risk of potassium imbalances in DKA, we conducted a retrospective cohort study to evaluate the efficacy of potassium replenishment and to explore the risk factors associated with hypokalemia.

## Methods

### Study design and setting

The participants in this study were recruited from seven tertiary central hospitals spanning the east, the central, and the west China regions, including the Affiliated Jiangning Hospital of Nanjing Medical University, Suqian First Hospital, the Affiliated Suqian Hospital of XuZhou Medical University (Nanjing Drum Tower Hospital Group Suqian Hospital), the Third Hospital Affiliated with Nanchang University, the Third People’s Hospital of Bengbu, Liuzhou People’s Hospital, and the People’s Hospital of Laibin. The selection period spanned from1 January 2021 to 31 December 2023, and targeted patients admitted with DKA. Clinical and laboratory data were meticulously extracted from the medical records of patients who were admitted to the endocrinology and metabolism wards, general internal medicine wards, and intensive care units, encompassing cases with DKA of varying severities, from mild to severe. All centers managed hypokalemia according to the Chinese Guidelines for the Prevention and Treatment of Type 2 Diabetes (2020 Edition) and the reference (9). The potassium replenishment regimen for patients was detailed in the Appendix 1.

The study was approved by the Ethics Committee of the hospitals and it was registered in the China Clinical Trials Registry (Registration No. ChiCTR2300077025) (October 26, 2023) and performed according to the principles of the Declaration of Helsinki. As it was a retrospective study, written informed consent could not be received from all patients or guardians. Patient informed consent was obtained through verbal agreement during a telephone follow-up.

### Inclusion and exclusion criteria

We included all patients presenting with DKA, excluding those who had received potassium chloride (KCl) replenishment before admission, as such treatment could confound potassium measurements. An episode of DKA was defined as present if a patient’s plasma glucose was > 13.9 mmol/L, urinary ketones were rated + to +++, and the arterial pH was < 7.30. The DKA severity standard was as follows: pH < 7.1 was considered severe, 7.1 ≤ pH < 7.2 was considered moderate, and 7.2 ≤ pH < 7.3 was considered mild. Potassium concentration was classified as follows: hypokalemia (< 3.5 mmol/L), normokalemia (3.5-5.2 mmol/L), and hyperkalemia (> 5.2 mmol/L) (10). The exclusion criteria and study flowchart have been shown in **Supplementary Figure 1**.

### Clinical data collection

Peripheral venous blood samples were collected for the following biochemical measurements according to standardized procedures: plasma glucose, glycated hemoglobin (HbA1c), serum creatinine (Scr), serum potassium (K^+^), sodium (Na^+^), chloride (Cl^-^), total calcium (TCa^2+^), phosphorus (P), magnesium (Mg^2+^), arterial blood gas analysis, and other clinical and biochemical indicators. All biochemical tests in this study were routine clinical assays performed according to established standard operating procedures.

Electrolytes were measured daily to capture hypokalemia. The potassium concentration measured at the completion of replenishment was recorded as the final concentration-listed in the Results table as “Last K^+^ after replenishment”. Hypokalemia at admission and during treatment were defined differently. In normokalemia and hyperkalemia groups, hypokalemia during treatment refered to the lowest serum potassium concentration recorded during the management process. However, the occurrence of hypokalemia during treatment in the hypokalemia group was defined as any further reduction in serum potassium below the already-low admission concentration. The serum anion gap was calculated by subtracting chloride and bicarbonate from sodium. The estimated glomerular filtration rate (eGFR) was calculated as described in the reference (11). Effective osmotic pressure (mmol/L) = 2 × Na^+^ (mmol/L) + glucose (mmol/L). The amount of KCl supplemented was calculated from the oral and intravenous sources (12).

### Statistical analysis

SPSS (version 26.0), R software (version 4.2.1) and RStudio (version 2021.09.02) were used for statistical analysis. For two-group comparisons, the t-tests was utilized for assessing normally distributed data, whereas for nonnormally distributed data, Mann–Whitney U tests was applied. Categorical data comparisons were made via the chi-square test or Fisher’s exact test, as appropriate. For three-group comparisons, the Kruskal–Wallis test was utilized for assessing nonnormally distributed data, ANOVA was utilized for assessing normally distributed data.

All the participants were randomly assigned to the training and validation groups at a 7:3 ratio. Both univariate and multivariable logistic regression analyses were performed, subsequently, a forward–backward stepwise selection procedure (*p* ≤ 0.05) was applied to identify significant risk factors and construct the nomogram model. The ‘rms’ package was utilized for nomogram construction and calibration curve plotting.

The model’s predictive performance was evaluated via metrics such as the area under the ROC curve, the Hosmer–Lemeshow test, and analysis of the calibration curve. Decision curve analysis (DCA) was employed to assess the model’s clinical utility, and internal validation was conducted to ensure the reliability and consistency of the model.

## Results

### Study population characteristics

Among the 654 participants enrolled, 571 (87.3%) eligible subjects were included in the study, including 172 with T1D and 399 with T2D. The cohort included 237 females and 334 males. Upon admission, the patients exhibited significantly elevated levels of glucose and HbA1c, as well as significantly reduced levels of arterial pH and HCO_3_^−^ (**Table 1**). Among the 571 participants, 48 patients with critical status were admitted to the ICU because of severe infections, acute abdomen, acute pancreatitis, and shock. The remaining patients were treated in the general ward, with severe DKA managed in the resuscitation room of the general ward. Admission to the ICU and the resuscitation room was not solely due to hypokalemia. The precipitating factors and comorbidities of the patients were listed in the Appendix 2.

**Table 1:** Comparison of characteristics and electrolyte levels between T1D and T2D patients with DKA.

Variables	Overall population (n = 571)	T1D (n = 172)	T2D (n = 399)	*P*-value
Female, n (%)	237 (41.5)	87 (50.6)	150 (37.6)	0.004
Age (years)	39.7 ± 17.6	28.6 ± 14.0	44.5 ± 16.8	<0.001
Course of diabetes (years), M (P_25_, P_75_)	2 (0, 7)	4 (1, 7)	1 (0, 7)	<0.001
Plasma glucose (mmol/L)	28.5 ± 11.1	28.2 ± 11.7	28.7 ± 10.8	0.63
HbA1c (%)	12.0 ± 2.7	12.2 ± 2.9	11.9 ± 2.5	0.16
Arterial blood pH	7.11 ± 0.14	7.10 ± 0.15	7.11 ± 0.14	0.57
HCO_3_^-^ (mmol/L)	8.1 ± 4.7	7.4 ± 4.3	8.0 ± 4.4	0.22
K^+^ at admission (mmol/L)	4.5 ± 1.0	4.5 ± 1.0	4.4 ± 1.0	0.27
Hypokalemia, n (%)	95 (16.6)	26 (15.1)	69 (17.3)	0.52
≤ 2.0 mmol/L, n	3	1	2	
2.1 - 2.5 mmol/L, n	10	4	6	
2.6 - 2.9 mmol/L, n	10	2	8	
3.0 - 3.5 mmol/L, n	72	20	52	
Hyperkalemia, n (%)	124 (21.7)	42 (24.4)	82 (20.6)	0.30
5.2 - 5.5 mmol/L, n	48	16	32	
5.6-5.9 mmol/L, n	30	8	22	
≥ 6.0 mmol/L, n	46	16	30	
KCl replenishment (days)	4.5 ± 2.7	4.1 ± 2.6	4.7 ± 2.8	0.01
KCl replenishment (g/d)	4.7 ± 2.5	4.6 ± 2.1	4.8 ± 2.6	0.39
Last K^+^ after replenishment (mmol/L)	3.8 ± 0.5	3.8 ± 0.5	3.8 ± 0.5	0.74
Hypokalemia during treatment, n (%)	388 (67.9)	111 (64.5)	277 (69.4)	0.19
Hypokalemia < 3.0 mmol/L during treatmentn, n (%)	205 (35.9)	56 (32.6)	149 (37.3)	0.27
Na^+^ (mmol/L)	134.8 ± 7.4	141.8 ± 7.5	141.1 ± 8.1	0.32
TCa^2+^ (mmol/L)	2.1 ± 0.4	2.1 ± 0.4	2.1 ± 0.4	0.74
Mg^2+^(mmol/L)	0.9 ± 0.2	0.8 ± 0.2	0.9 ± 0.2	0.08
Cl^-^ (mmol/L)	100.8 ± 8.9	101.4 ± 8.0	100.6 ± 9.2	0.33
P (mmol/L)	1.2 ± 0.9	1.3 ± 1.0	1.1 ± 0.8	0.003
Scr (μmol/L)	94.2 ± 64.5	77.2 ± 46.1	99.3 ± 62.4	<0.001
eGFR (mL/min per 1.73 m^2^)	118.3 ± 77.9	141.8 ± 82.0	108.3 ± 74.0	<0.001
Anion gap (mmol/L)	35.5 ± 14.8	34.9 ± 14.0	35.8 ± 15.2	0.54
Effective osmotic pressure (mmol/L)	323.8 ± 24.8	324.1 ± 26.1	323.8 ± 24.2	0.88

Data are presented as mean ± SD or median (P_25_, P_75_), *P* values represent results of t-tests or Mann–Whitney U test for continuous variables or the χ^2^ or Fisher's exact test for categorical variables.

### DKA management and potassium replenishment

Immediately initiate emergency treatment upon admission for DKA: first, gave rapid fluid resuscitation with 0.9% sodium chloride, adjusting the volume to the degree of dehydration to restore circulating volume. Started a continuous intravenous insulin infusion at 0.1 unit/kg/h to lower glucose gradually. For potassium repletion, followed guidelines and and the reference (9): supplement potassium chloride daily, monitor serum potassium closely to prevent hypokalemia. If pH < 7.0, cautiously administered small doses of sodium bicarbonate to correct acidosis. Concurrently, we treated any co-existing conditions such as respiratory or urinary infections, coma, palpitations, or trauma to improve overall outcome.

As the patients’ condition improved, blood glucose levels stabilized, ketones were cleared, osmotic diuresis subsided, and food intake normalized. Once serum potassium K^+^ concentration fell within the normal range of 3.5-5.0 mmol/L, potassium supplementation was gradually tapered. The daily dose of KCl was reduced to less than 3.0 g. Potassium supplementation was continued for an additional 1–3 days before being discontinued.

### Differences between T1D and T2D

Significant differences were observed between T1D and T2D groups. Compared with T1D group, T2D group was older and required longer duration of KCl replenishment, had higher serum creatinine levels, and exhibited a lower proportion of females, shorter diabetes duration, reduced eGFR, and lower serum phosphorus levels (all *p* < 0.05). However, there were no significant differences between T1D and T2D patients in terms of other parameters (**Table 1**).

### Differences between mild - moderate and severe DKA

In **Table 2**, the patients were classified according to the severity of DKA, with 340 cases categorized as mild to moderate DKA and 231 cases as severe DKA. Significant differences were observed in glucose, HbA1c, and K^+^ levels between the mild to moderate and severe DKA groups (all *p* < 0.01). The lower pH and HCO_3_^-^ in severe versus mild to moderate DKA reflect the severity criteria themselves and add no clinical insight. Five individuals succumbed to severe DKA, accounting for 0.9% of the total cohort, two had hypokalaemia on admission, whereas the remaining three maintained normokalemia. Serum potassium concentration at admission varied widely among patients: 95 had concentration below 3.5 mmol/L, 352 were within the range of 3.5 to 5.2 mmol/L, and 124 exceeded 5.2 mmol/L (**Table 3**).

**Table 2:** Comparison of characteristics and electrolyte levels between mild to moderate and severe DKA patients.

Variables	Mild to moderate DKA (n = 340)	Severe DKA (n = 231)	*P*-value
Female, n (%)	134 (39.4)	102 (44.2)	0.24
Age (years)	40.8 ± 17.5	38.1 ± 17.5	0.07
Course of diabetes (years), M (P_25_, P_75_)	2.5 (0, 6)	2 (0, 8)	0.64
Plasma glucose (mmol/L)	26.6 ± 9.6	31.4 ± 12.4	<0.001
HbA1c (%)	11.7 ± 2.6	12.3 ± 2.7	0.01
Arterial blood pH	7.20 ± 0.06	6.96 ± 0.09	<0.001
HCO_3_^-^ (mmol/L)	9.7 ± 4.0	4.7 ± 3.1	<0.001
K^+^ at admission(mmol/L)	4.3 ± 0.9	4.7 ± 1.2	<0.001
Hypokalemia, n (%)	61 (17.9)	34 (14.7)	0.31
≤ 2.0 mmol/L, n	1	2	
2.1 - 2.5 mmol/L, n	5	5	
2.6 - 2.9 mmol/L, n	9	1	
3.0 - 3.5 mmol/L, n	46	26	
Hyperkalemia, n (%)	55 (16.2)	69 (29.9)	<0.001
5.2 - 5.5 mmol/L, n	29	19	
5.6-5.9 mmol/L, n	12	18	
≥ 6.0 mmol/L, n	17	29	
KCl replenishment (days)	4.4 ± 2.6	4.6 ± 2.9	0.10
KCl replenishment (g/d)	4.5 ± 2.2	5.1 ± 2.7	0.004
Last K^+^ after replenishment (mmol/L)	3.9 ± 0.4	3.7 ± 0.5	0.001
Hypokalemia during treatment, n (%)	218 (64.1)	170 (73.6)	0.01
Hypokalemia < 3.0 mmol/L during treatmentn, n (%)	102 (30.0)	103 (44.6)	<0.001
Na^+^ (mmol/L)	140.7 ± 7.5	142.2 ± 8.6	0.03
TCa^2+^ (mmol/L)	2.1 ± 0.4	2.0 ± 0.4	0.07
Mg^2+^(mmol/L)	0.8 ± 0.2	0.9 ± 0.2	0.002
Cl^-^ (mmol/L)	100.5 ± 8.2	101.3 ± 9.8	0.31
P (mmol/L)	1.0 ± 0.6	1.4 ± 1.2	0.001
Scr (μmol/L)	84.0 ± 52.3	105.5 ± 65.4	<0.001
eGFR (mL/min per 1.73 m^2^)	128.6 ± 78.2	102.8 ± 75.1	<0.001
Anion gap (mmol/L)	33.0 ± 13.3	39.4 ± 16.2	<0.001
Effective osmotic pressure (mmol/L)	320.0 ± 23.6	329.8 ± 25.5	<0.001

Data are presented as mean ± SD or median (P_25_, P_75_), *P* values represent results of t-tests or Mann–Whitney U test for continuous variables or the χ^2^ or Fisher's exact test for categorical variables.

**Table 3:** Comparison of characteristics among hypokalemia, normokalemia and hyperkalemia of DKA patients at admission.

Variables	Hypokalemia (n = 95)	Normokalemia (n = 352)	Hyperkalemia (n = 124)	*P*-value
Female, n (%)	49 (51.6)	141 (40.1)	46 (37.1)	0.07
Age (years)	35.6 ± 16.8	39.8 ± 17.8	41.7 ± 17.0*	0.04
Course of diabetes(years), M (P_25_, P_75_)	1 (0, 7)	2 (0, 7)	4 (0, 7)	0.06
Plasma glucose (mmol/L)	26.1 ± 9.6	27.4 ± 10.8	33.8 ± 11.3**††	<0.001
HbA1c (%)	12.6 ± 2.4	12.0 ± 2.7	11.5 ± 2.6*	0.01
Arterial blood pH	7.12 ± 0.14	7.12 ± 0.14	7.05 ± 0.13**††	<0.001
HCO_3_^-^ (mmol/L)	8.1 ± 4.0	8.5 ± 4.5	5.6 ± 3.5**††	<0.001
K^+^ at admission (mmol/L)	3.1 ± 0.4	4.3 ± 0.5**	5.9 ± 0.7**††	<0.001
KCl replenishment (days)	5.2 ± 2.8	4.4 ± 2.7*	4.2 ± 2.6*	0.01
KCl replenishment (g/d)	6.3 ± 3.2	4.5 ± 2.2**	4.0 ± 1.9**	<0.001
Last K^+^ after replenishment (mmol/L)	3.7 ± 0.5	3.8 ± 0.4*	3.9 ± 0.6*	0.006
Hypokalemia during treatment, n (%)	93 (97.9)	228 (64.8)**	67 (54.0)**	<0.001
Na^+^ (mmol/L)	141.5 ± 7.6	141.3 ± 8.1	141.1 ± 7.8	0.95
TCa^2+^ (mmol/L)	2.0 ± 0.3	2.1 ± 0.4	2.2 ± 0.4**†	0.001
Mg^2+^(mmol/L)	0.8 ± 0.2	0.9 ± 0.2**	0.9 ± 0.2**	<0.001
Cl^-^ (mmol/L)	105.0 ± 8.1	101.3 ± 8.6**	96.5 ± 8.5**††	<0.001
P (mmol/L)	0.7 ± 0.4	1.1 ± 0.7**	1.7 ± 1.2**††	<0.001
Scr (μmol/L)	66.7 ± 48.6	88.5 ± 53.7*	124.6 ± 66.6**††	<0.001
eGFR (mL/min per 1.73 m^2^)	165.8 ± 94.6	121.0 ± 74.2**	74.5 ± 44.6**††	<0.001
Anion gap (mmol/L)	32.8 ± 17.4	34.7 ± 15.2	40.8 ± 8.9*†	0.001
Effective osmotic pressure (mmol/L)	318.6 ± 23.1	323.2 ± 25.6	330.2 ± 22.7*†	0.002

Data are presented as mean ± SD or median (P_25_, P_75_), *P* values represent results of ANOVA or Kruskal-Wallis tests for continuous variables or the χ^2^ or Fisher's exact test for categorical variables. Comparison with hypokalemia group: (*) *p* < 0.05, (**) *p* < 0.001, comparison with normokalemia group (†) *p* < 0.05, ( ††) *p* < 0.001.

During treatment, hypokalemia developed in 111 T1D patients, representing 64.5% of the total T1D DKA patients. Among T2D patients, 277 experienced hypokalemia, which was 69.4% of the total T2D patients (**Table 1**). Specifically, 218 patients with mild to moderate DKA developed hypokalemia during treatment, comprising 64.1% of this subgroup. The incidence of hypokalemia was significantly higher among patients with severe DKA (*p* = 0.01), affecting 170 patients (73.6% of the severe DKA group) (**Table 2**).

In **Table 2**, patients with severe DKA were admitted with higher serum potassium concentration (4.7 ± 1.2 mmol/L) compared to those with mild to moderate DKA (4.3 ± 0.9 mmol/L) (*p* < 0.001). Additionally, the mean daily potassium supplementation was slightly higher in severe DKA group (5.1 ± 2.7 g/d) than in mild to moderate DKA group (4.5 ± 2.2 g/d) (*p* = 0.004). The incidence of serum potassium concentration falling below 3.0 mmol/L during treatment was significantly higher in severe DKA (44.6%) than that in mild to moderate DKA (30.0%) (*p* < 0.001).

### Differences among hypo-, hyper-, and normokalemia

In **Table 3**, the hyperkalemia group exhibited a higher mean age, compared with normokalemia and hypokalemia groups, patients who were admitted with hypokalemia required more potassium supplementation (*p* < 0.001). With an increased mean daily supplementation of 6.3 ± 3.2 g KCl during treatment, recurrent hypokalemia was observed in 97.9% of the hypokalemia DKA population. The incidence of hypokalemia during treatment was 64.8% and 54.0% in normokalemia and hypokalemia groups, respectively. Hyperkalemia was associated with eGFR (r = -0.419, *p* < 0.001) and age (r = 0.149, *p* < 0.001).

### Logistic regression analysis

**Figure 1:**
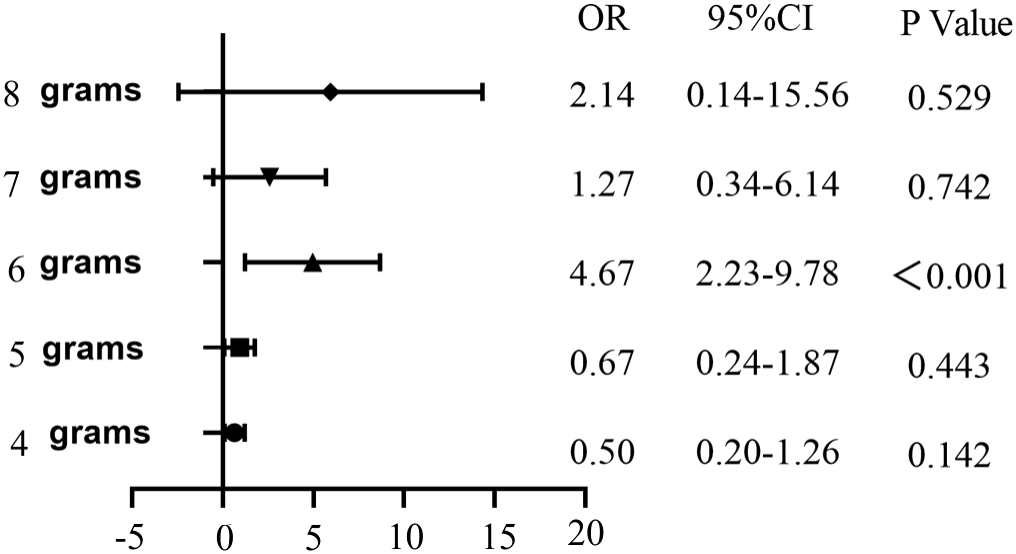
Univariate logistic regression analysis of the occurrence of hypokalemia in DKA patients with different daily potassium supplementation.

In this study, both univariate and multivariate logistic regression analyses were employed to examine the clinical variables detailed in **Table 4**. The daily amount of potassium supplementation was found to be closely related to the incidence of hypokalemia during treatment (OR = 1.42, 95% CI: 1.23-1.63, *p* < 0.001). The incidence of hypokalemia varied significantly depending on the daily amount of KCl supplementation. Univariate logistic regression analysis revealed a significant correlation between supplementation with 6.0 g/d KCl and the occurrence of hypokalemia (OR = 4.67, 95% CI: 2.23-9.78, *p* < 0.001) (**Figure 1**).

**Table 4:** Univariate and multivariate logistic regression analyses for hypokalemia during treatment in patients with DKA.

Variables	Univariate		Multivariate	
	OR (95% CI)	*P-*value	OR (95% CI)	*P-*value
Gender (n)	1.48 (0.92-2.40)	0.07		
Age (years)	0.98 (0.97-1.00)	0.007		
Diabetes classification	1.05 (0.64-1.75)	0. 84		
K^+^ at admission (mmol/L)	0.56 (0.44-0.72)	<0.001	0.55 (0.41-0.74)	<0.001
Na^+^ (mmol/L)	1.02 (0.99-1.06)	0.23		
Cl^-^ (mmol/L)	1.03 (1.00-1.06)	0.02		
Arterial blood pH	0.11 (0.02-0.62)	0.01	0.05 (0.01-0.49)	0.01
Scr (umol/L)	1.00 (0.99-1.00)	0.07		
Plasma glucose (mmol/L)	1.00 (0.98-1.02)	0.94		
HbA1c (%)	1.15 (1.05-1.27)	0.003	1.11 (1.01-1.23)	0.04
Average daily potassi- um supplement (g)	1.42 (1.23-1.63)	<0.001		
Days of potassium supplementation (d)	1.51 (1.31-1.73)	<0.001	1.49 (1.29-1.72)	<0.001
Six grams potassium daily	4.67 (2.23-9.78)	<0.001	4.03 (1.79-9.06)	0.001
Total supplemental potassium (g)	1.10 (1.07-1.13)	<0.001		

In **Table 4**, the forward-backward logistic regression analysis identified that K^+^ concentration at admission, HbA1c, pH, 6.0 g/d KCl, and the days of potassium supplementation were all independently associated with the occurrence of hypokalemia during treatment, with significant correlations (*p* < 0.05). Specifically, HbA1c, daily 6.0 g KCl replenishment, and the days of KCl supplementation were found to be risk markers for hypokalemia, while K^+^ concentration at admission and arterial pH were identified as protective factors against hypokalemia.

### Predictive model and nomogram

**Figure 2:**
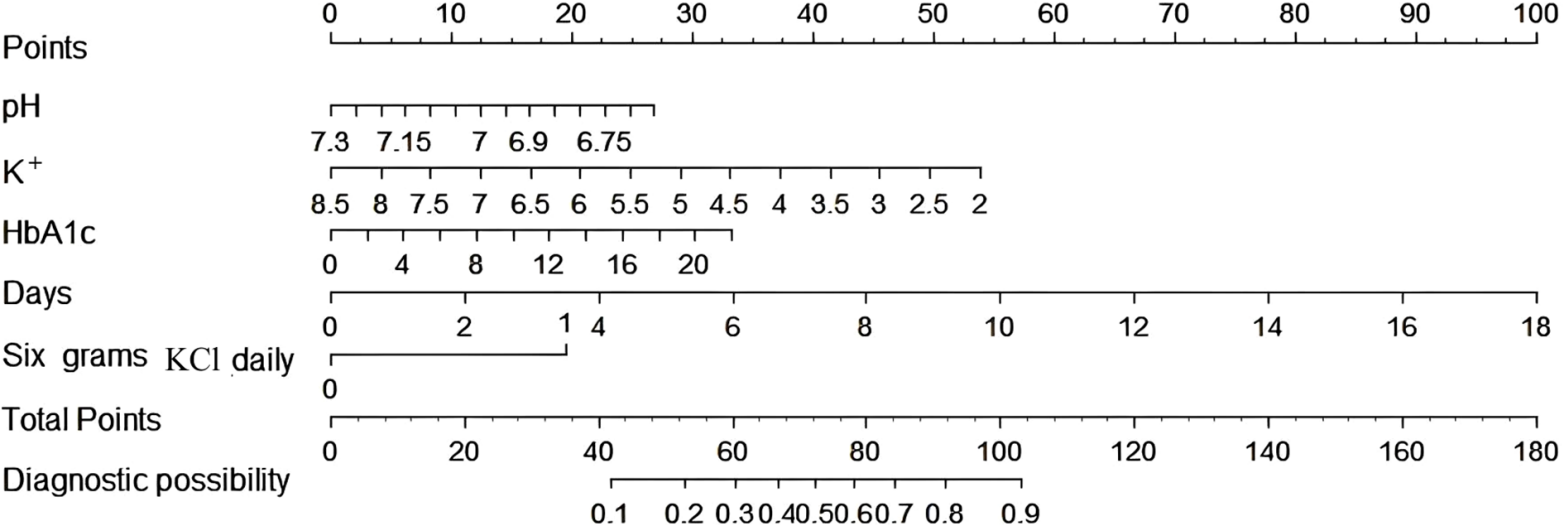
The nomogram model for quantifying individual risk of hypokalemia during DKA treatment.

Based on these findings, a predictive model was developed and visualized as a nomogram (**Figure 2**). The nomogram highlights that 6.0 g KCl per day and a moderate number of supplementation days are significant predictors of hypokalemia during treatment. Each predictor was assigned a score according to a specific scale within the nomogram, which corresponded to its respective contribution to hypokalemia. These scores were then summed to obtain a total score, which was used to estimate the likelihood of hypokalemia.

**Figure 3:**
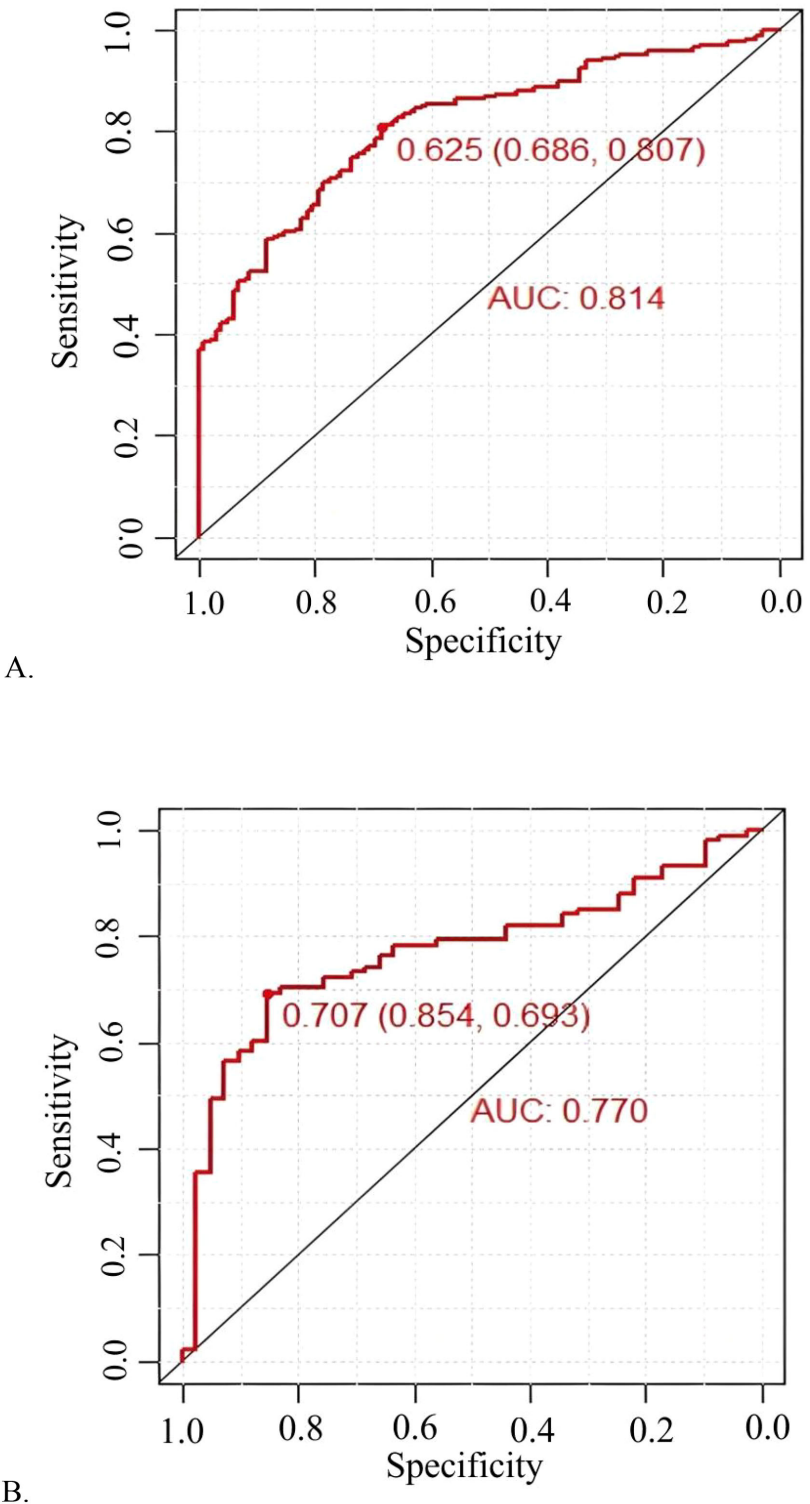
Prediction performance of the model. Receiver operating characteristic (ROC) curve plot in the training cohort **(A)**; ROC curve plot in the validation cohort **(B)**; AUC, the area under the ROC.

**Figure 4:**
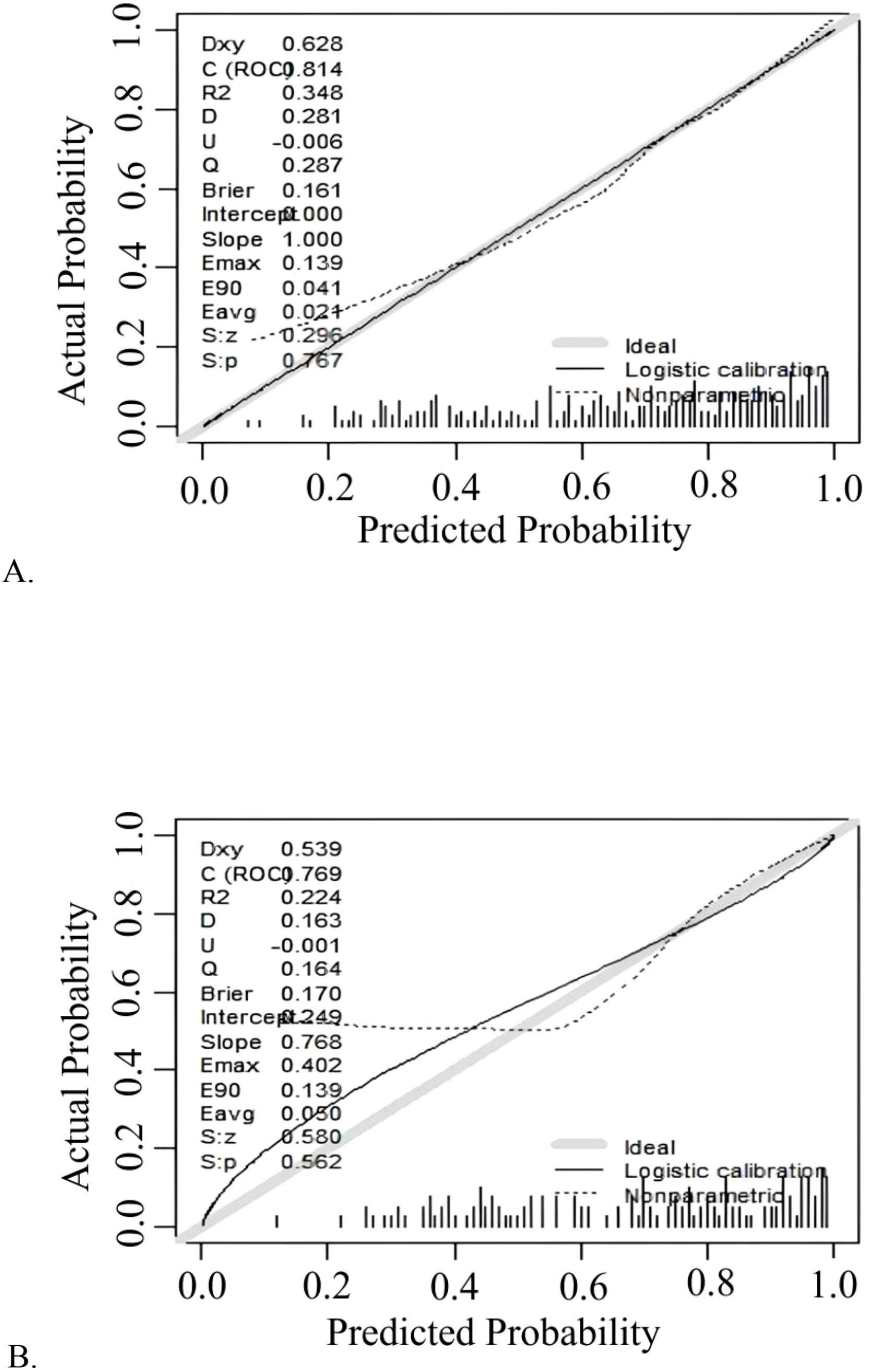
Calibration curve plot in each cohort. **(A)** the training cohort; **(B)** the validation cohort.

**Figure 5:**
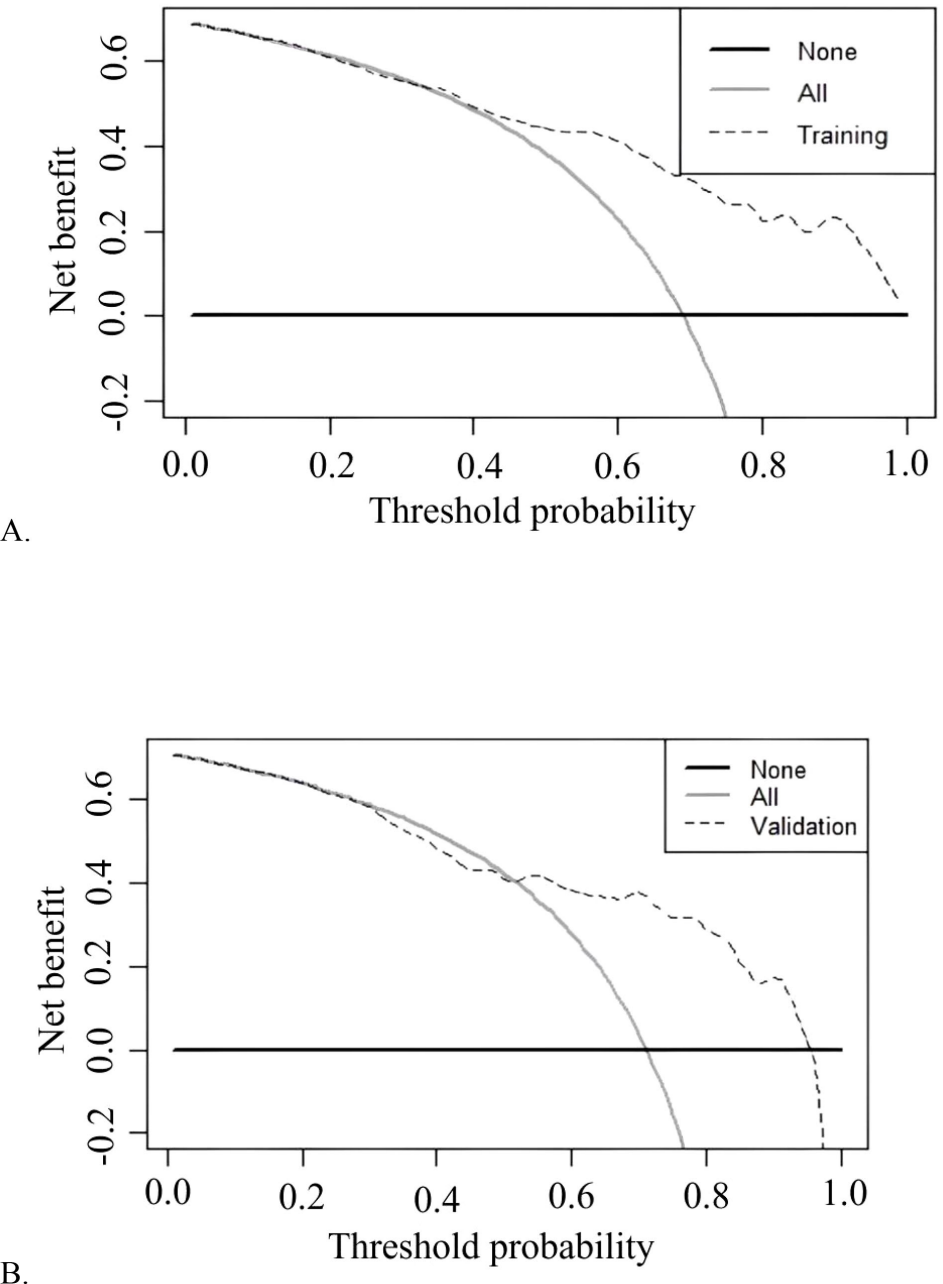
Decision curve analysis of training cohort **(A)** and validation cohort **(B)** for the risk of hypokalemia during DKA treatment.

The model demonstrated robust discriminatory power, with an AUC of 0.814 (95% CI: 0.768-0.860) in the training cohort (**Figure 3A**) and 0.770 (95% CI: 0.689-0.850) in the validation cohort (**Figure 3B**). The calibration plots, for both the training and validation cohorts, depicted in **Figure 4**, illustrated a strong alignment between the estimated probabilities from the model and the actual outcomes. The model’s calibration was further assessed using the Hosmer-Lemeshow (HL) test, which yielded a chi-square value of 14.85 (*p* = 0.10) for the training set and of 13.22 (*p* = 0.15) for the validation set, indicating a reliable model fit. The calibration curve for the training cohort provided an indication of how well the model’s predictions align with the actual outcomes within the data used to train the model. The calibration curve for the validation cohort evaluated how well the model performs in a separate dataset, thereby testing its generalizability to new, unseen data. As shown in **Figure 5**, the DCA results highlighted the clinical utility of the nomogram in predicting the risk of hypokalemia during treatment.

## Discussion

### Summary of the key findings

DKA patients presented with significant internal environmental disruptions, including hyperglycemia, metabolic acidosis, dehydration, and electrolyte imbalances. Hyperkalemia was observed more frequently than hypokalemia, and higher potassium concentration were associated with the severity of DKA, hypokalemia often arose during DKA management. We demonstrated that the daily KCl supplementation was generally inadequate. Furthermore, we developed a statistical model to predict the risk of hypokalemia during treatment, which could serve as a valuable tool for clinicians. A daily supplement of 6.0 g KCl was viewed as a marker of potassium deficit and DKA severity, signaling the increased potassium replenishment.

### DKA-related hypokalemia

DKA-related hypokalemia is an important but difficult to anticipate event, and its transient course can easily be overlooked. Patients with DKA are often characterized by significant potassium depletion, primarily due to absolute or relative insulin deficiency and the osmotic diuretic effect, which leads to the excretion of potassium along with ketone anions. Furthermore, the reduction in circulating volume caused by osmotic diuresis, coupled with secondary aldosteronism and gastrointestinal losses, can precipitate hypokalemia (4). As shown in this study, 95 (16.6%) were hypokalaemic, 352 (61.6%) normokalemic and 124 (21.7%) hyperkalaemic. Serum potassium concentration at admission varied depending on the timing of presentation, with some patients exhibiting hyperkalemia.

DKA is an acute, dynamic, and unpredictable emergency, and its mechanism of hypokalemia is complex. Primary aldosteronism, Gitelman syndrome, Bartter syndrome, and Liddle syndrome all present with hypokalemia, but their underlying mechanisms are relatively straightforward, representing a chronic process of potassium loss. The condition can often be managed with targeted treatments. In contrast, hypokalemia in DKA is fundamentally different. When we administer insulin to correct hyperglycemia, provide fluids to restore volume, and correct acidosis, hypokalemia can develop quietly and rapidly. It is far more difficult to prevent hypokalemia in DKA compared to the aforementioned conditions (13, 14).

### DKA-associated hyperkalemia

DKA-associated hyperkalemia is commonly caused by multiple underlying pathophysiologic mechanisms. Insulin deficiency, metabolic acidosis, and the accumulation of ketone bodies prevent potassium from entering cells, leading to its accumulation in the extracellular fluid. Dehydration resulting from DKA leads to hemoconcentration. Elevated stress hormones, such as catecholamines, further exacerbate hyperkalemia as they promote the efflux of potassium from cells. Although osmotic diuresis may lead to potassium loss, patients typically experience transient hyperkalemia at presentation due to the combined effects (4). In this study, hyperkalemia was observed more commonly than hypokalemia on admission, and higher potassium concentration were associated with the severity of DKA. Based on the pathophysiology of DKA, we observed that patients presenting with severe DKA had significantly higher admission serum potassium concentration than those with mild-to-moderate DKA (**Table 2**). The relative hyperkalemia reflected a shift of intracellular potassium into the extracellular compartment due to severe acidemia and insulin deficiency. In this study, when hyperkalemia was present, all centers ceased potassium supplementation, which was consistent with the guidelines followed by previous studies (2, 15). Consequently, when insulin therapy was initiated, extracellular potassium enters the cell, the development of hypokalemia was almost an inevitable outcome (16, 17).

### Misleading aspects of hyperkalemia and normokalemia

However, the initial hyperkalemia and normokalemia can be misleading. The early hyperkalemia masks a substantial potassium deficit, which is proportionally greater in severe DKA because of prolonged osmotic diuresis and vomiting. Insulin therapy, which is crucial for treating DKA, rapidly shifts potassium into cells, leading to a significant drop in serum potassium levels. This interpretation why, even after KCl supplementation, patients with severe DKA may still present with serum K^+^ concentration below 3.0 mmol/L. Therefore, careful monitoring and timely potassium supplementation are essential to prevent hypokalemia related complications in DKA management (4).

### Importance and strategy of potassium replenishment

Patients with DKA should receive potassium replenishment during the second hour of treatment unless they exhibit hyperkalemia or anuria. In adults, the absence of early potassium replenishment could lead to cardiac conduction issues, such as cardiac dysrhythmias (6). In pediatric patients with DKA, 31% exhibited an elongated QTc interval on their electrocardiograms, with the severity of DKA and the progression of acidosis correlating to a greater extent of QTc prolongation (18). Our retrospective design covered few continuous ECG and adjudicated arrhythmia data. Electrolyte shifts during acidemia correction may have produced transient signals. Future prospective, synchronized monitoring of electrolytes, acid-base, and cardiac electrophysiology is needed to detect the oscillations.

The primary cause of death in DKA was multifactorial, and hypokalemia is not the main cause of mortality. The literature had documented the adverse cardiac effects of hypokalemia (19, 20). Among the five fatal cases in our study, three had normokalemia, while two exhibited hypokalemia. This underscores that hypokalemia was not the primary cause of mortality. In addition, adequate potassium supplementation can shorten the length of hospital stay (21).

Current clinical guidelines recommend adjusting the dosage of potassium supplementation on the basis of the serum potassium concentration. However, there was a scarcity of evidence regarding the optimal dosage of potassium supplementation and its effectiveness. Prior research had examined electrolyte imbalances, with a focus on potassium, and had revealed that potassium supplementation was often inadequate (4, 12). During the course of treatment, 388 out of 571 DKA patients developed hypokalemia, accounting for 67.9% of the total, which was higher than the 55% rate reported by Pasquel FJ et al. (22). The higher proportion of patients with hypokalemia might be associated with poor adherence to the protocol guidance on potassium supplementation (23).The high incidence of hypokalemia or hyperkalemia in this study highlighted the complex interplay between initial hyperkalemia due to acidemia and insulin deficiency, and subsequent rapid declines in serum potassium following insulin therapy, particularly in severe DKA.

### Interpretation of the predictive model

In this study, we observed that hypokalemia occurred during management was common, which was consistent with previous research (12, 19, 22). The positive association between daily potassium dose and hypokalemia appears counterintuitive. Potassium supplementation was initiated before hypokalemia occurred. Therefore, the subsequent development of hypokalemia reflected an insufficient dose to counteract the characteristic insulin-driven intracellular shift of potassium in DKA.

We conducted univariate logistic regression analyses of different daily amounts of potassium supplementation and found that there was a significant correlation between daily supplementation with 6.0 g of KCl and the occurrence of hypokalemia. Therefore, we established a nomogram for predicting hypokalemia during DKA treatment. The analysis identified K^+^ concentration at admission, HbA1c, pH, 6.0 g KCl daily, and number of days of KCl supplementation as key independent predictors of hypokalemia during treatment.

As for pH, in the process of DKA, hyperglycemia and ketoacidosis induced osmotic diuresis increases potassium excretion, contributing to potassium depletion in intracellular. Usman A et al. also indicated that pH adjusted potassium concentration significantly affect hypokalemic changes on an electrocardiogram (15). Lower HbA1c levels were associated with reduced ketoacidosis risk (24, 25), arteria pH and HbA1c returned a negative correlation, a higher HbA1c was associated with more severe DKA (26). Severe DKA often have relative hyperkalemia initially, but they also had larger total K^+^ deficits, which was consistent with our research. Although the exact mechanism was unclear, it could be concluded that ketoacidosis, inflammation and oxidative stress and other factors caused an impairment of renal excretion (8).

We aimed to provide a suggestion for managing potassium disorders in DKA, expecting initial improvements in clinical outcomes and reduced hypokalemia related cardiovascular risks. Daily 6.0 g KCl and KCl supplementation days were fundamental measures for basic intervention. We hope this striking finding will attract the attention of our peers. Galm et al. and Usman et al. also indicated that hypokalaemia should be ameliorated with active potassium supplementation (12, 16). Regardless of whether patients presented with hypo-, hyper-, or normokalemia, guideline-based replenishment strategies alone were insufficient to prevent subsequent disturbances, underscoring the need for individualized DKA potassium management (5). The recommended 6 g of KCl per day was a starting dose, not a ceiling. The nomogram provided by the authors allowed rapid bedside estimation of individual hypokalemia risk. If ongoing potassium losses persisted-osmotic diuresis, vomiting, or intracellular shift after correction of alkalosis-serial monitoring of serum potassium was required and the potassium supplementation dose must be titrated accordingly.

### Limitations

Retrospective multicenter DKA data were valuable yet limited: record quality, recall and centre-specific care could bias sample and information, and residual confounders might distort findings. Nonetheless, the large real-world cohort furnished reliable estimates of potassium metabolism patterns.

## Conclusions

In this study, DKA on admission was characterized by severe internal biochemical disruptions. Potassium disorders were highly prevalent. Hyperkalemia was more prevalent than hypokalemia at admission. Additionally, hypokalemia occurred during management was common. Potassium supplementation was frequently found to be inadequate. Although the model’s estimates of optimal daily KCl replenishment remained theoretical. Daily 6.0 g KCl, a mirror of DKA severity, did not mean higher doses provoke hypokalemia. Managing potassium deficiency in DKA was complex. Determining the precise potassium dose remained challenging. Future research should focus on ensuring that protocols incorporate the current evidence-based practices to optimize patient clinical outcomes.

## Data Availability

The raw data supporting the conclusions of this article will be made available by the authors, without undue reservation.

## Supplementary material

Supplementary Figure 1 The exclusion criteria and study flowchart.
