# Supplementary figures and images for: Real-world insights from acute management of potassium disorders in diabetic ketoacidosis
Source: Front Endocrinol (Lausanne). 2025 Nov 3;16:1669400. doi: 10.3389/fendo.2025.1669400 (PMC12620269; doi:10.3389/fendo.2025.1669400)

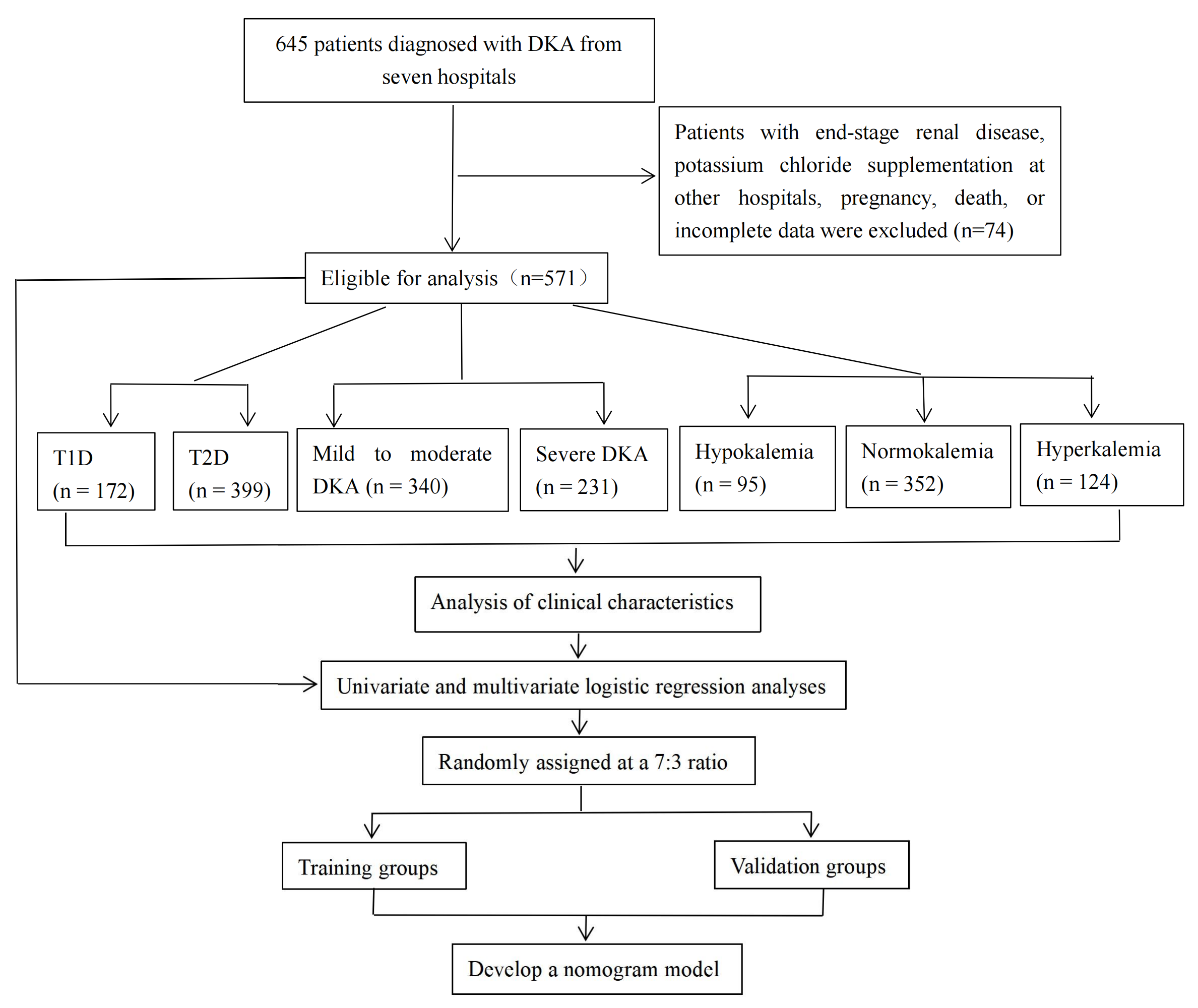

Supplement: Supplementary Figure 1 — The exclusion criteria and study flowchart. [file Image1.tif]
