# Supplementary material for: Real-world insights from acute management of potassium disorders in diabetic ketoacidosis
Source: Front Endocrinol (Lausanne). 2025 Nov 3;16:1669400. doi: 10.3389/fendo.2025.1669400 (PMC12620269; doi:10.3389/fendo.2025.1669400)
Supplement: Supplementary file 2 [file DataSheet1.zip › Appendix/Appendix 1.docx]

Potassium supplementation protocols

• K^+^ > 5.5 mmol/L: no supplementation is required owing to the risk of precipitating cardiac arrhythmias with additional potassium.

• K^+^ 4.0-5.0 mmol/L: 20mmol/L of replacement fluid.

• K^+^ 3.0-4.0 mmol/L: 40mmol/L of replacement fluid.

• K^+^ ≤ 3.0-4.0 mmol/L: 10-20 mmol per hour until K^+^ > 3.0 mmol/L, then add 40 mmol/L to replacement fluid.

Note: 20mmol/L of replacement fluid equivalent to 1.5 g KCl/L substitution fluid.

Refs:

[1] Chinese Diabetes Society of the Chinese Medical Association.Chinese Guidelines for the Prevention and Treatment of Type 2 Diabetes 2020 Edition (in Chinese). Zhonghua Tang Niao Bing Za Zhi. 2021,13(4):315-409.DOI:10.3760/cma.j.cn115791-20210221-00095.

[2] Galm BP, Bagshaw SM, Senior PA. Acute Management of Diabetic Ketoacidosis in Adults at 3 Teaching Hospitals in Canada: A Multicentre, Retrospective Cohort Study. Can J Diabetes. 2019 Jul;43(5):309-315.e2. doi: 10.1016/j.jcjd.2018.11.003.

[3] Dhatariya KK, Glaser NS, Codner E, Umpierrez GE. Diabetic ketoacidosis. Nat Rev Dis Primers (2020) 6:40. doi: 10.1038/s41572-020-0165-1
