# Supplementary material for: Real-world insights from acute management of potassium disorders in diabetic ketoacidosis
Source: Front Endocrinol (Lausanne). 2025 Nov 3;16:1669400. doi: 10.3389/fendo.2025.1669400 (PMC12620269; doi:10.3389/fendo.2025.1669400)
Supplement: Supplementary file 2 [file DataSheet1.zip › Appendix/Appendix 2.docx]

Appendix 2 A. Precipitating factors of hyperglycemia.

|  | T1D  (n=172) | T2D  (n=399) |
| --- | --- | --- |
| Inadequate treatment | 68 | 85 |
| Newly diagnosed diabetes | 23 | 93 |
| Insulin discontinuation | 8 | 14 |
| Sweet foods | 6 | 11 |
| Infections | 24 | 48 |
| Other idiopathic causes | 43 | 148 |

Appendix 2 B. Comorbidities of the DKA patients.

|  | T1D  (n=172) | T2D  (n=399) |
| --- | --- | --- |
| Acute abdomen | 124 | 214 |
| Shock, palpitations | 13 | 47 |
| Coma | 9 | 39 |
| Acute pancreatitis | 2 | 5 |
| Infections | 24 | 48 |
| Others | 25 | 51 |
