# Supplementary material for: Real-world insights from acute management of potassium disorders in diabetic ketoacidosis
Source: Front Endocrinol (Lausanne). 2025 Nov 3;16:1669400. doi: 10.3389/fendo.2025.1669400 (PMC12620269; doi:10.3389/fendo.2025.1669400)
Supplement: Supplementary file 2 [file DataSheet1.zip › Appendix/Appendix Table 3.docx]

**Appendix Table 3.** Comparison of serum potassium by gender

| Variables | Female DKA  (n = 236) | Male DKA  (n =335) | *p*-value |
| --- | --- | --- | --- |
| Hypokalemia, n (%) | 49 | 46 | 0.026 |
| Normokalemia, n (%) | 141 | 211 | 0.43 |
| Hyperkalemia, n (%) | 46 | 78 | 0.30 |
